# Lessons from a case of osteopetrosis oxycephaly and Chiari type I malformation: a case report

**DOI:** 10.4076/1757-1626-2-6787

**Published:** 2009-07-27

**Authors:** Aimun AB Jamjoom, Bakur A Jamjoom, Abrar R Waliuddin, Abdulhakim B Jamjoom

**Affiliations:** 1 University of Nottingham, Faculty of Medicine and Health Sciences, Medical SchoolQueen’s Medical Centre, Nottingham NG7 2UHUK; 2 Section of Neurosurgery, King Khalid National Guards HospitalJeddahSaudi Arabia

## Abstract

We report a child with osteopetrosis, oxycephaly and Chiari type I malformation who presented with raised intracranial pressure. During cranial expansion surgery the patient developed sudden cardiac arrest which we believe was probably related to the Chiari malformation. The case highlights a previously unrecognized association between osteopetrosis, craniosynostosis and a persistently open fontanelle at age 4 years. In addition it supports the existing literature in emphasizing the need for careful preoperative work up, choice of approach and operative technique in children with complex craniosynostosis and Chiari malformation.

## Introduction

Osteopetrosis is a genetically determined bone disease that develops as a result of malfunction of osteoclastic activity leading to excessive deposition of immature bone, thickening of cortical bones and narrowing of the medullary cavities [1]. The resulting bone thickening of calverium and skull base are responsible for the recognized number of cranial and intracranial manifestations of the disease [2]. Craniosynostosis embraces a group of developmental diseases that include oxycephaly and are usually associated with early closure of sutures and anterior fontanelle. We report an unusual child with osteopetrosis, oxycephaly and Chiari type I malformation who presented with raised intracranial pressure (ICP). The aim of the presentation is to document a previously unrecognized occurrence of osteopetrosis, oxycephaly and a persistently open fontanelle at age 4 years. The case also supports the existing literature regarding the need for a careful preoperative work up, choice of approach and technique in children with complex craniosynostosis and Chiari malformation.

## Case presentation

A 4-year-old Saudi Arabian Middle Easterner male presented to our neurosurgical unit with an abnormally shaped head, stunted growth, developmental delays, blindness with recurrent headaches and opisthotonus posturing. He was diagnosed elsewhere as a case of infantile osteopetrosis and had a brother that died from complications related to the disease. His other three siblings were normal. On examination he was small for age (weight 10.5 kg and height 84.5 cm) and able to communicate with limited vocabulary. His head was small (circumference 45 cm), conical in shape with an open anterior fontanelle and prominent scalp veins. He had bilateral optic atrophy and no bulbar palsy. He was unsteady on his feet with increase tone in limbs but no weakness.

**Figure 1.**
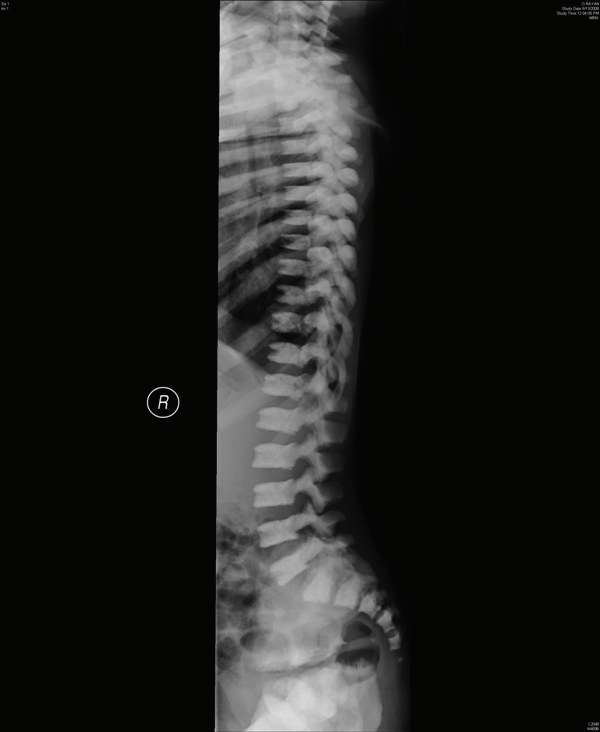
Plain radiograph of spine showing the hyperstotic vertebral endplates.

**Figure 2.**
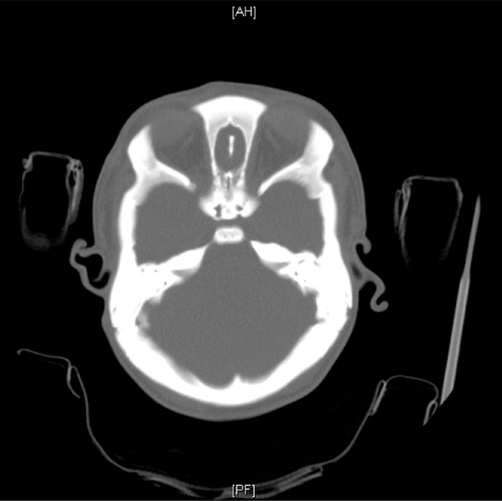
CT head (3-D reconstruction) showing oxycephaly with bone growth around an open fontanelle and hypertrophy of the metopic suture posteriorly and the coronal sutures medially.

**Figure 3.**
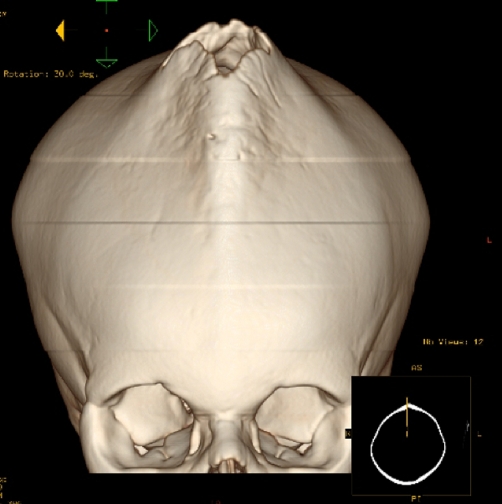
CT Brain showing bony sclerosis and thickening of the skull base due to osteopetrosis.

**Figure 4.**
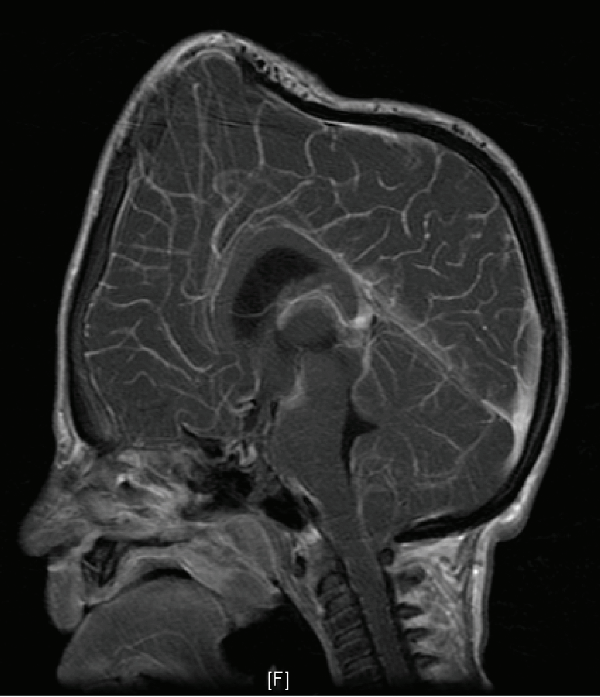
MRI (TI sagittal with contrast) showing Chiari type 1 malformation, upward bulging of frontal lobes towards the bregma, shallow anterior cranial fossa and extensive subcutaneous venous channels.

Haemoglobin was 12.5 g/dl and serum urea and electrolytes and calcium were normal. Skeletal survey showed evidence of bone sclerosis throughout the axial and appendicular skeleton with metaphyseal widening and endobone formation at vertebral end plates (Figure 1). Computed tomography (CT) (Figure 2) showed a conical head with considerable bone growth around an open fontanelle and hypertrophy of the metopic suture posteriorly and the coronal sutures medially. There was also evidence of bony sclerosis and thickening of the skull base (Figure 3), narrowing of optic foramina and shortening of anterior cranial fossa with exorbitism but no hypertelorism or mid face hypoplasia. In addition the patient’s magnetic resonance imaging (MRI) (Figure 4) showed Chiari type I malformation, upward bulging of frontal lobes towards the bregma with extensive venous channels crossing the skull and poor visualization of the superior sagittal sinus at that level. Magnetic resonance angiography (MRA) was reported normal.

We felt that the patient's main clinical problem was raised ICP related to reduction in the intracranial volume rather than the Chiari malformation. We therefore elected to offer the patient in the first instance cranial expansion surgery in the form of bifrontal craniotomy, orbital advancement and excision of hypertrophied bone at the bregma. At surgery there were numerous venous channels between the scalp, pericranium and epidural space particularly at the level of the open fontanelle. Despite taking appropriate care to control bleeding, the estimated blood loss up to the lifting of the bone flap was 300 mls for which the patient received 2 units of packed red blood cells. The patient’s blood pressure (BP) was maintained at 100/60 and pulse of 100/minute however, following the completion of the bifrontal craniotomy and in the absence of evidence of excessive bleeding or change in oxygenation, pulse rate or end tidal CO2 values, the patient’s BP unexpectedly dropped to 60/40 and within a few minutes he went into cardiac arrest. All efforts at resuscitation failed and the patient was pronounced dead. Autopsy permission was not granted.

## Discussion

The clinical characteristics, family history, laboratory and imaging studies suggest that our patient was suffering from the autosomal recessive (malignant) osteopetrosis and that at the time of surgery he had not developed the full manifestations of the disease. Patients with this entity are known to exhibit poor osseous growth and remodeling, anaemia, infection and haemorrhage as a result of obliteration of the marrow spaces. Other manifestations include developmental delay, short stature, cranial nerve palsies, optic atrophy, narrowing of skull base foramina, hypocalcemia, renal tubular acidosis and frequent fractures. Benign autosomal dominant and intermediate autosomal recessive forms of the disease are also recognized [1,2].

The optic atrophy in our child is likely to be primary optic atrophy related to optic foramen narrowing rather that a consequence of raised ICP. His skull was affected by a number of localized synostoses and the shape was most compatible with type III false oxycephaly [3]. The latter is a late-appearing craniosynostosis that is associated with a high risk of ophthalmic and mental complications [4]. We feel justified in labeling the skull abnormality of our patient as oxycephaly despite the presence of an open fontanelle. It is recognized that the anterior fontanelle may remain open in a healthy child up to 32 months of age [5]. The association of a persistently open fontanelle at age 4 years with osteopetrosis and craniosynostosis as in our patient is previously unrecognized in the literature.

From the pathogenesis point of view, the osteopetrosis and oxycephaly in our patient are likely to be unrelated. In osteopetrosis the osteoclasts dysfunction affects cartilage clearance, which reduces marrow spaces in long bones and leads to extramedullary hematopoiesis and because of reduced bone clearance results in “bone-in-bone formation”. Since membranous bone does not have cartilage, the irregular bone deposition does not occur and the skull demonstrates radiologically a smooth generalized bone thickening from failure of bone resorption rather than focally over suture sites [1]. On the other hand sutural fusion is modulated by anti-growth factors rather than osteoclastic activity [6].

Patients with oxycephaly commonly have raised ICP and venous hypertension due venous drainage abnormality particularly at the sigmoid-jugular complex [7]. The intracranial hypertension may develop after an aesthetically successful cranial vault expansion in around third of patients with syndromic synostosis [8]. In addition, the effects of the venous hypertension may last until the affected child is approximately 6 years old [9]. The bone thickening in osteopetrosis may also cause raised ICP and venous hypertension as a result of reduction in intracranial volume and narrowing of the jugular foramen. This would explain why our patient has raised ICP that was manifested by headaches, open fontanelle at 4 years, congested scalp veins and numerous intracranial-extracranial venous channels crossing the skull on MRI. The latter could have been delineated better by a preoperative magnetic resonance venography (MRV) study. Our patient did not have hydrocephalus. It is recognized that abnormal cerebrospinal fluid (CSF) hydrodynamics can be found in 8% of craniosynostosis patients [10].

Chiari malformation is rarely seen with osteopetrosis [11] but is a frequent finding in multisutural and syndromic craniosynostoses occurring in 75% of patients with oxycephaly [7]. The type I malformation can develop rapidly in the face of raised ICP, craniosynostosis and spinal CSF diversion [12]. The pathogenesis of this condition and the rationale for treatment remains controversial. However, it is thought that the malformation is an acquired and progressive condition that develops early in life because of a disproportion between hindbrain growth and the abnormally small posterior fossa, a consequence of premature fusion of cranial base sutures, rather than primary malformation of brain development [7,13]. The cranio-cephalic mismatch, the osteopetrosis-related limitation in intracranial volume, the raised ICP and venous hypertension in our patient are likely to enhance the development and/or progression of the brain deformation. Such explanation would support our decision to offer cranial expansion surgery for the oxycephaly first rather than a posterior fossa expansion or a foramen magnum decompression. Naturally a more accurate assessment of the raised ICP could have been obtained by a period of ICP monitoring. Thompson et al [13] reported that the levels of raised ICP correlated significantly with the extent of hindbrain herniation and the smallness of the posterior fossa size.

Chiari type I malformation can present with a wide variety of clinical symptoms that can be attributed to bulbar and/or medullary distress, compression of the midbrain ascending reticular system or vascular compromise (vertebrobasilar artery compression) [14]. Rare but recognized Chiari malformation presentations include orthostatic intolerance and syncope [14] and sudden death [15]. The increase tone in limbs, unsteadiness on feet and the opisthotonus posturing could have been related to the Chiari malformation [16] and may have been an indication to decompress the foramen magnum first.

Our child, who had Chiari type I malformation, suffered an unexplained cardiac arrest during cranial expansion surgery for oxycephaly. Such intraoperative arrest is more often the result of sudden hypovolaemic shock or embolism but the blood loss had been replaced in our patient and the clinical picture was not supportive of an embolic cause. We have considered that the lifting of the large bone flap may have caused a rapid drop in blood pressure due to sudden relief of the raised ICP [17]. However, we believe that the arrest was related to the Chiari malformation and that it was caused by the Chiari-related medullary dysfunction that may have been exacerbated by the operative blood loss.

We feel that the Chiari-related cardiac arrest of our patient supports the need for the neurosurgeon to consider decompressing the posterior fossa first in patients with craniosynostosis and Chiari malformation. However, as these patients are likely to have abnormal intracranial-to-extracranial venous drainage, a preoperative MR or CT venogram should be performed to demonstrate any widely dilated veins at the craniocervical junction which may influence the decision to do the surgery [18,19]. In addition efforts at reducing blood loss by the use of diathermy to incise the scalp or the use of cell saver autologous blood transfusion should be considered [20]. The case also supports the need for care during the lifting of the bone flap to avoid sudden relief of the raised ICP.

## Conclusions

The case highlights a previously unreported association between osteopetrosis, craniosynostosis and a persistently open fontanelle at age 4 years. It also supports the existing literature in emphasizing the importance of careful preoperative planning and identification of the vasculature in complex craniosynostosis patients. Based on the clinical picture and imaging studies the neurosurgeon should consider decompressing the posterior fossa first in patients with craniosynostosis and Chiari malformation. Care in replacing blood loss and in avoiding sudden drop in ICP are necessary.

## Abbreviations

BP: blood pressure
CO2: carbon dioxide
CSF: cerebrospinal fluid
CT: computed tomography
ICP: intracranial pressure
MRA: magnetic resonance angiography
MRI: magnetic resonance imaging
MRV: magnetic resonance venography
